# Supplementary material for: Remote Neuroinflammation in Newly Diagnosed Glioblastoma Correlates with Unfavorable Clinical Outcome
Source: Clin Cancer Res. 2024 Aug 16;30(20):4618–34. doi: 10.1158/1078-0432.CCR-24-1563 (PMC11474166; doi:10.1158/1078-0432.CCR-24-1563)
Supplement: Supplementary Data 1 — Supplementary Figures 1, 2 Supplementary Tables 1 - 3 inlucing Representativeness of Study Participants" supplementary table (Supplementary Table 1) [file ccr-24-1563_supplementary_data_1_suppds1.pdf]

Supplement

Supplementary Figure 1

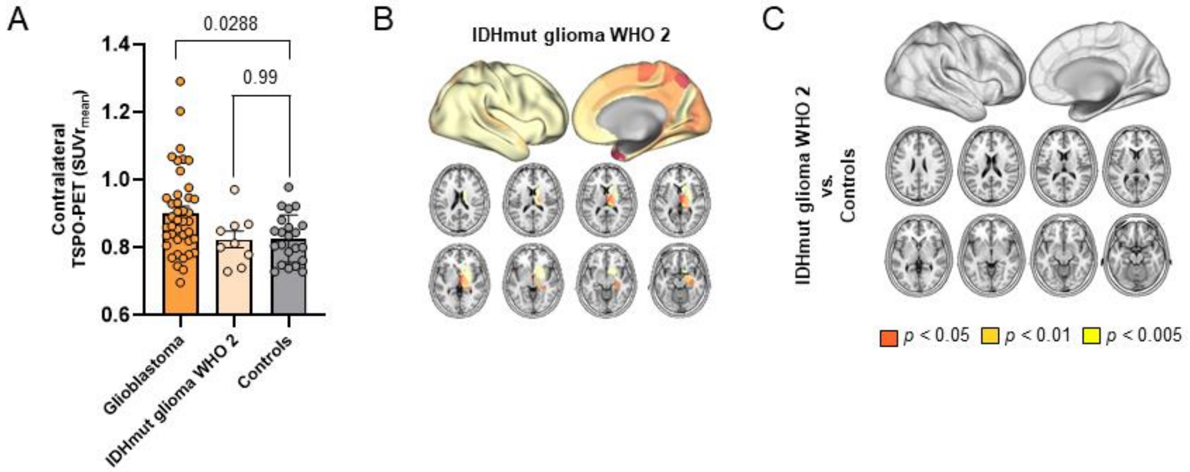

Supplementary Figure 1: Assessment of TSPO-PET signals in the non-lesional hemisphere of patients with IDHmut glioma WHO 2.

(A) Patients with newly diagnosed IDHmut glioma WHO 2 (n=7 astrocytoma, n=2 oligodendroglioma) do not indicate higher TSPO-PET signal in the contralateral hemisphere compared to healthy controls.

(B) Surface projections and axial slices of the group average contralateral TSPO-PET signal of patients with IDHmut glioma WHO 2. The non-lesional hemispheres were parcellated into 123 sub-regions using the Brainnetome Atlas. Axial slices show TSPO-PET signals of basal ganglia regions (masked cortical regions).

(C) No significant TSPO-PET signal elevation in brain regions of the contralateral hemisphere could be detected in patients with IDHmut glioma WHO 2 compared to healthy controls.

Supplementary Figure 2

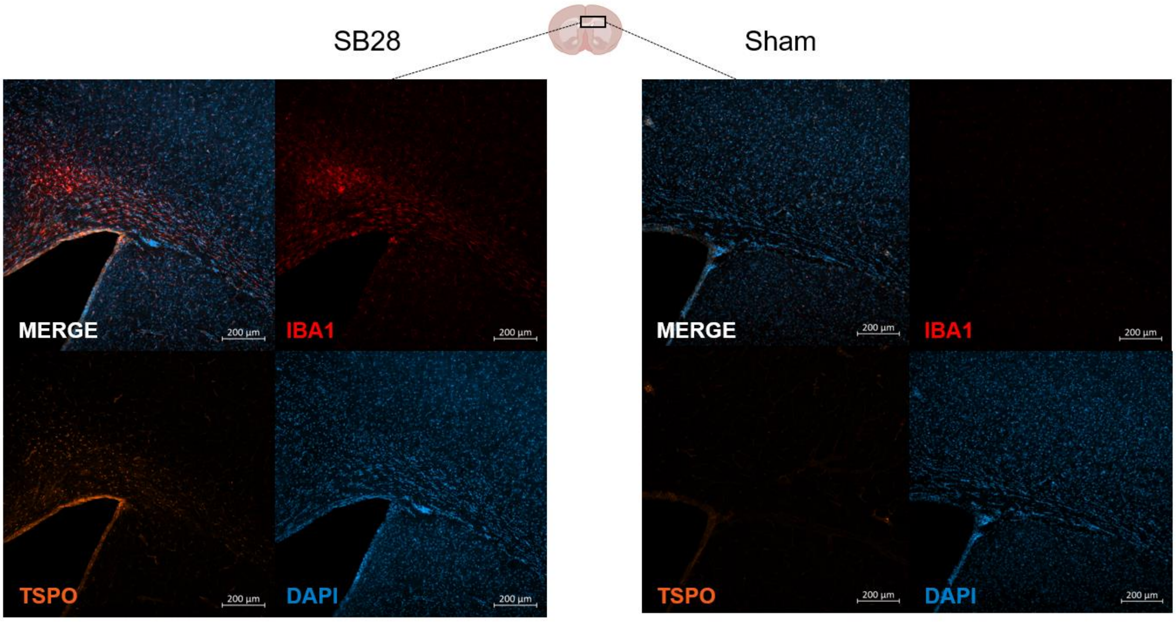

Supplementary Figure 2: Qualitative Immunofluorescence of the corpus callosum in SB28 glioblastoma mice.
Coronal slices of immunofluorescence of a SB28 glioblastoma mouse show high abundance of IBA1/TSPO-positive cells in the corpus callosum of the contralateral hemisphere.

Supplementary Table 1

	Median overall survival (range)*	Significance**
**Age**		**p = 0.015**
Median < 72.6y, n=16	14.28 (1.50-57.17)	
Median > 72.6y, n=15	6.53 (0.53-21.37)	
**Sex**		p = 0.953
Male, n=21	10.83 (1.50-57.17)	
Female, n=10	11.40 (0.53-43.73)	
**SNP**		p = 0.777
LAB, n=5	10.53 (4.17-19.93)	
MAB, n=16	11.58 (0.53-57.17)	
HAB, n=10	7.17 (0.93-27.67)	
**MGMT promoter methylation status**		p = 0.581
Methylated, n=13	11.57 (0.53-57.17)	
Unmethylated, n=18	10.43 (0.93-27.67)	
**TERT promoter mutation status**		p = 0.352
mutant, n=26	10.83 (0.53-43.73)	
wildtype, n=4	19.43 (10.53-57.17)	
**Glucocorticoid therapy**		p = 0.053
yes, n=10	5.17 (0.53-25.77)	
no, n=20	11.58 (3.07-57.17)	
**Tumor resection**		p = 0.152
yes, n=7	19.07 (1.50-57.17)	
no, n=24	10.28 (0.53-43.73)	
**Radiotherapy**		**p = 0.024**
yes, n=27	11.60 (0.93-57.17)	
no, n=4	5.30 (0.53-11.57)	
**Chemotherapy**		**p = 0.004**
yes, n=20	13.88 (4.03-57.17)	
no, n=11	5.17 (0.53-25.77)	
**Temozolomide**		p = 0.306
yes, n=14	13.40 (4.17-57.17)	
no, n=17	9.10 (0.53-43.73)	
**Tumor CE volume (cm3)**		**p < 0.001**
Median < 8.0, n=16	12.65 (4.17 - 57.17)	
Median > 8.0, n=15	6.53 (0.53 - 27.67)	
**Tumor T2 volume (cm3)**		**p < 0.001**
Median < 60.1, n=16	15.34 (0.53 - 57.17)	
Median > 60.1, n=15	9.10 (0.93 - 21.37)	
**Tumor TSPO-PET signal (SUVr)**		**p = 0.024**
Median < 2.5, n=16	13.58 (0.53 - 57.17)	
Median > 2.5, n=15	9.10 (0.93 - 27.67)	
**Contralateral TSPO-PET signal (z score)**		**p < 0.001**

Median < 0.5, n=16	13.40 (0.53 - 57.17)	
Median > 0.5, n=15	6.53 (0.93 - 27.67)	

Supplementary Table 1: Univariate survival analysis. Univariate Cox regression was used to test for significance of distinct parameters and indices on patient survival. SNP = single nucleotide polymorphism. LAB = ow, MAB = medium, HAB = high affinity binding status. MGMT = O-6-Methylguanine-DNA methyltransferase. TERT = Telomerase reverse transcriptase. CE = contrast enhancement. *time indicated in months. **bold font highlights statistically significant associations.

Supplementary Table 2

	Hazard ratio (95% CI)	Significance*
**Age**	1.123 (1.055 - 1.196)	**p < 0.001**
**MGMT promoter methylation status**	0.199 (0.058 - 0.684)	**p = 0.010**
**Glucocorticoid therapy**	5.162 (1.450 - 18.370)	**p = 0.011**
**Radiotherapy**	0.274 (0.059 - 1.286)	p = 0.101
**Chemotherapy**	0.689 (0.251 - 1.896)	p = 0.471
**Tumor CE volumes (cm3)**	1.039 (1.005 - 1.074)	**p = 0.025**
**Tumor TSPO-PET signal (SUVr)**	0.709 (0.286 - 1.760)	p = 0.459
**Contralateral TSPO-PET signal (z score)**	2.175 (1.263 - 3.744)	**p = 0.005**

Supplementary Table 2: Multivariate survival analysis. Multivariate Cox regression was used to test for significance of relevant (significant in univariate Cox regression) parameters and indices on patient survival. Glucocorticoid medication was additionally included due to borderline significance. CE = contrast enhancement. MGMT = O-6-Methylguanine-DNA methyltransferase. MGMT was additionally included due to significance in an extended cohort. *bold font highlights statistically significant associations.

Supplementary Table 3

	**Hazard ratio (95% CI)**	**Significance***
**Age**	1.204 (1.080 - 1.342)	**$p < 0.001$**
**Sex**	1.111 (0.261 - 4.693)	$p = 0.886$
**SNP**	0.295 (0.089 - 0.984)	**$p = 0.047$**
**MGMT promoter methylation status**	0.017 (0.001 - 0.196)	**$p = 0.001$**
**TERT promoter mutation status**	0.421 (0.134 - 1.318)	$p = 0.137$
**Glucocorticoid therapy**	14.135 (1.595 - 125.302)	**$p = 0.017$**
**Tumor resection**	0.253 (0.051 - 1.268)	$p = 0.095$
**Radiotherapy**	0.086 (0.007 - 1.088)	$p = 0.058$
**Chemotherapy**	1.215 (0.241 - 6.122)	$p = 0.814$
**Tumor CE volumes (cm3)**	1.061 (1.010 - 1.115)	**$p = 0.019$**
**Tumor T2 volumes (cm3)**	1.004 (0.979 - 1.030)	$p = 0.757$
**Tumor TSPO-PET signal (SUVr)**	0.378 (0.100 - 1.428)	$p = 0.151$
**Contralateral TSPO-PET signal (z score)**	2.010 (1.105 - 3.656)	**$p = 0.022$**

Supplementary Table 3: Extended multivariate survival analysis. Multivariate Cox regression was used to test for significance of all considered parameters and indices on patient survival. CE = contrast enhancement. MGMT = O-6-Methylguanine-DNA methyltransferase. *bold font highlights statistically significant associations.
